# Genomic Instability Associated with p53 Knockdown in the Generation of Huntington’s Disease Human Induced Pluripotent Stem Cells

**DOI:** 10.1371/journal.pone.0150372

**Published:** 2016-03-16

**Authors:** Andrew M. Tidball, M. Diana Neely, Reed Chamberlin, Asad A. Aboud, Kevin K. Kumar, Bingying Han, Miles R. Bryan, Michael Aschner, Kevin C. Ess, Aaron B. Bowman

**Affiliations:** 1 Department of Neurology, Vanderbilt University Medical Center, Nashville, TN, 37240, United States of America; 2 Vanderbilt Brain Institute, Vanderbilt University, Nashville, TN, 37232, United States of America; 3 Genetics Associates Inc., Nashville, TN, 37203, United States of America; 4 Department of Molecular Pharmacology, Albert Einstein College of Medicine, Bronx, NY, 10461, United States of America; 5 Department of Pediatrics, Vanderbilt University Medical Center, Nashville, TN, 37240, United States of America; 6 Center in Molecular Toxicology, Vanderbilt University, Nashville, TN, 37232, United States of America; University of Minnesota Medical School, UNITED STATES

## Abstract

Alterations in DNA damage response and repair have been observed in Huntington’s disease (HD). We generated induced pluripotent stem cells (iPSC) from primary dermal fibroblasts of 5 patients with HD and 5 control subjects. A significant fraction of the HD iPSC lines had genomic abnormalities as assessed by karyotype analysis, while none of our control lines had detectable genomic abnormalities. We demonstrate a statistically significant increase in genomic instability in HD cells during reprogramming. We also report a significant association with repeat length and severity of this instability. Our karyotypically normal HD iPSCs also have elevated ATM-p53 signaling as shown by elevated levels of phosphorylated p53 and H2AX, indicating either elevated DNA damage or hypersensitive DNA damage signaling in HD iPSCs. Thus, increased DNA damage responses in the HD genotype is coincidental with the observed chromosomal aberrations. We conclude that the disease causing mutation in HD increases the propensity of chromosomal instability relative to control fibroblasts specifically during reprogramming to a pluripotent state by a commonly used episomal-based method that includes p53 knockdown.

## Introduction

Huntington’s disease (HD) neuropathology results from neuronal death of neurons primarily in the caudate and putamen of the basal ganglia. Although this neuronal degeneration has been well-characterized, the mechanism by which mutant Huntingtin protein expression leads to cell death is not well understood. Elevated phosphorylation of DNA damage signaling pathway markers such as p53 is a hallmark of HD, and variants in the gene encoding p53 (*TP53*) modify the age of onset in HD patients [1, 2]. Mutant Huntingtin expressing cells have also been shown to be sensitive to X-rays and resistant to immortalization by the T-antigen, further implicating alterations in DNA damage response pathway and p53 signaling in HD [3, 4]. Recent studies have shown altered DNA repair kinetics of important double-strand break (DSB) repair proteins, such as Ku70 and MRE11, and binding or alterations in activity of the critical DNA damage signaling proteins p53 and ataxia telangiectasia mutated (ATM) in HD cell and animal models [1, 5–11]. Further evidence for altered DNA damage response and repair is derived from observations that, unlike their healthy relatives, HD patients have a significantly decreased risk of nearly all forms of cancer versus national incidence rates [12].

Human induced pluripotent stem cells (iPSCs) have a high propensity for developing genomic abnormalities, which is likely due to the “uncoupling” of checkpoint activation and apoptosis [13]. Additionally, many common chromosomal abnormalities in human embryonic stem cells (ESCs) and iPSCs provide a growth advantage, such as trisomy 12 [14]. While performing normal validation of newly generated iPSC lines, we discovered a significantly higher occurrence of genomic abnormalities in iPSC lines generated from HD patients compared to control lines. In this study, we investigated both the time at which the abnormalities arose in culture (fibroblast culture, reprogramming, or iPSC culture) and the mechanistic source of genomic instability. We found that abnormalities most likely occurred during the reprogramming time period and that karyotypically normal HD iPSC lines also demonstrated elevated DNA damage signaling. These data further indicate underlying alterations in DNA damage and/or repair processes in HD cells.

## Materials and Methods

### Generation of human iPSC lines

Human dermal fibroblasts were obtained at Vanderbilt University Medical Center (CA, CC, CD, CE, C-ESS, HD35, HD57, and HD58) and from Coriell Cell Biorepository (catalog.coriell.org) (GM21756 and GM09197 which are called here, HD70 and HD180 respectively). Primary dermal fibroblasts were obtained by skin biopsy from healthy adult subjects (CA, CC, CD, CE and C-ESS) with no known family history of neurodegenerative disease and HD patients (HD35, HD57, and HD58) after obtaining donor (or parental) written consent for using the sample for research purposes under the guidelines of a Vanderbilt University approved IRB protocol (Vanderbilt #080369). Cells were grown to ~ 80% confluency and harvested by trypsinization. 6x10^5 cells were then electroporated with the pCXLE plasmid vectors using the Neon Transfection System (Life Technologies, Carlsbad, CA), according to the conditions described in Okita et al 2011 [15]. Plasmids pCXLE-hOCT3/4-shp53-F, pCXLE-hSK, and pCXLE-hUL were from Dr. Shinya Yamanaka (Addgene plasmid # 27077, 27078, and 27079, respectively).

Cells were replated in DMEM with 10% FBS and grown with daily media changes for 7 days. The cells were then trypsinized and replated at 1x10^5 cells per 100 mm dish on top of SNL feeder cells. The next day, the medium was changed to KOSR ES medium (described in [16]) and changed daily for ~30 days before colonies were picked and propagated. For the HD35 and HD57 lines, 50,000 cells were electroporated with the pCXLE plasmids and directly replated on Matrigel (BD Biosciences, San Jose, CA) coated 6 well plates in DMEM with 10% FBS. After 3 days, the medium was changed to TeSR-E7 and changed daily until colonies were picked into mTeSR1 medium (StemCell technologies, Vancouver, BC).

### Cell culture

iPSC lines were maintained in mTeSR1 medium on Matrigel coated 6-well plates. Media was replaced daily. Cells were passaged at ~50% confluency. Before passage, spontaneously differentiated cells were manually removed with a specific home-made glass scraper [16]. Colonies were washed once in DMEM/F12 (Life Technologies, Carlsbad, CA) and incubated for 5–10 min with dispase (StemCell Technologies, Vancouver, BC). Colonies were then washed twice with Dulbecco’s Modified Eagle’s Medium (DMEM)/F12, scrapped into mTeSR1, triturated several times to achieve smaller cell clump size, and replated at a dilution of 3–5 fold onto Matrigel.

### Karyotype analysis

iPSC lines were submitted for standard g-band karyotype analysis by Genetics Associates, Inc., (Nashville, TN). For each line, 20 metaphase spreads were analyzed and normal lines as defined in this study were euploid in 20 of 20 cells. For “aged” iPSC and fibroblast experiments the number of metaphase spreads was increased to 100.

### Immunoblot analysis

Protein samples were prepared by scraping cells into ice-cold PBS, centrifugating the lysates, and resuspending the pellets in RIPA buffer containing protease (Sigma-Aldrich, St. Louis, MO) and phosphatase inhibitor cocktails 2 & 3 (Sigma, Sigma-Aldrich, St. Louis, MO). After gentle homogenization, cells were centrifuged at 4°C for 10 minutes at 20,000 *g*. The protein concentration of the supernatant was determined using the DC assay (BioRad, Hercules, CA). Samples were mixed with 5x SDS loading buffer containing 1% 2-mercaptoethanol and boiled for 5 minutes. 15 μg of protein was loaded for each sample onto a 4–15% pre-cast gel SDS-PAGE gel (BioRad, Hercules, CA) and run at 90V for 120 minutes. The proteins were transferred onto nitrocellulose membranes using iBlot Gel Transfer Device (Life Technologies). The proteins remaining in the gel after the transfer were stained with IRDye Blue protein stain (LI-COR, Lincoln, NE), imaged on a Li-Cor Odyssey Imaging System and proteins quantified. These values were used to normalize the values of immunostained bands. To validate our normalization technique, we compared total protein calculated by this method to actin band intensity and saw no differences. The membrane was blocked in Odyssey Blocking Buffer for one hour and then incubated in primary antibodies diluted in Odyssey Blocking Buffer containing 0.1% TWEEN 20 overnight at 4°C. See ref [11] for antibody dilutions. After washing 5 times for 5 minutes in Tris-buffered saline with 0.05% TWEEN 20, membranes were incubated with secondary antibodies at 1:10,000 (LiCor, Lincoln, NE) for 1 hour at room temperature. Membranes were imaged using the Li-Cor Odyssey Imaging System, and quantification was performed using Image Studio Lite (LiCOR, Lincoln, NE).

### In-cell western assay

Method described in Tidball et al, 2015 [11]. Plates were imaged and intensities quantified using the Li-Cor Odyssey Imaging System, and quantification was performed using Image Studio Lite (LiCOR, Lincoln, NE).

### MTT [3-(4,5-dimethylthiazol-2-yl)-2,5-diphenyltetrazolium bromide] assay

For the neocarzinostatin treatment viability assay, induced pluripotent stem cells were plated at 100,000 cells/mL in mTeSR1 with Rho-kinase inhibitor, Y-27632 (Tocris, Bristol, UK). Cells were exposed to neocarzinostatin in mTeSR1 for 1 hour followed by washing twice in phosphate-buffered saline (PBS) and replacing with fresh mTeSR1 medium. Cells were then placed back in the incubator for an additional 23 hours. 0.5% MTT in mTeSR1 was added to each well. After 2 hours of incubation, the medium was removed, and cellular formazan crystal dissolved in a solution of 10% Sorenson’s buffer and 90% dimethyl sulfoxide (DMSO). Absorbance was measured at 570–590 nm.

### Centrosome counting

iPSCs were grown on Nunc Lab-Tek I Chamber slides (Thermo Scientific). Cells were fixed in 4% paraformaldehyde in PBS for 30 min at room temperature, permeabilized with 0.2% Triton-X100 for 20 min at room temperature, and then incubated in PBS containing 5% donkey serum and 0.05% Triton-X100 for 2 h at room temperature or overnight at 4°C. Cell were incubated in Pericentrin (Abcam 1:1000) antibody overnight at 4°C, washed in PBS with 0.05% Triton-X100 and incubated for 3 hours with secondary antibody. Cells were mounted in Prolong Gold Antifade Reagent with 4',6-diamidino-2-phenylindole (DAPI) (Life Technologies). Cells undergoing mitosis were identified by loss of nuclear envelope and distinct chromosome morphology with a Zeiss ObserverZ1 microscope. Cells with 1 or 2 distinct pericentrin-positive centrosomes were scored as normal, while those with 3 or more were counted as abnormal. 20 cells were scored for each cell line in each experiment by an investigator blinded with regards to genotype.

### Cell cycle analysis

Control and HD iPSCs were treated for 1 hour with 2 ng/μL of neocarzinostatin in mTeSR1, washed twice with PBS, and incubated for 23 hours in mTeSR1 in a cell culture incubator at 37°C and 5% CO_2_. The cells were then dissociated with Accutase, washed once in cold PBS, and fixed in cold 70% ethanol for 30 min at 4°C. After 2 washes in PBS, the cells were treated with RNase and incubated with the DNA dye, propidium iodide (PI) for 10 minutes prior to analysis. The samples were analyzed on a Custom Becton Dickson five-laser Fortessa analytical cytometer using BD FACSDiva acquisition (BD Biosciences) and FlowJo analysis software (Tree Star, Inc., Ashland, OR). A total of 10,000 cells (events) were acquired. Analysis was restricted to single cells determined by gating using the side and forward light scatter properties. Percentages of cells in each cell cycle for each sample were determined based on the intensity of PI fluorescence (G0/1, S, and G2 in ascending order). Example histograms are displayed in S1 Fig.

### Statistical analysis

Statistical analysis was performed using either SPSS Statistics 22 (IBM) or Prism (GraphPad Software). Chi square test (Prism) was used to determine if disease state was a significant predictor of genomic instability. Two-way ANOVA (Prism) was used to analyze the disease and treatment effects for the western blot data, and repeated measures (for random subject effects) ANOVA (Prism) was used to determine the effect of concentration and disease for the iPSC MTT and In-Cell Western data. Sidak post-hoc test was used to determine significance of differences between sub groups with * representing p < 0.05. Paired t-test (Prism) was used to determine the effect of neocarzinostatin on cell cycle arrest. Binary logistic regression (SPSS) was performed using length of the longest *Huntingtin* CAG repeat, sex, age at biopsy, and passage number as fixed factors to determine whether they were significant predictors of genomic instability. We considered a p value < 0.05 as significant.

## Results and Discussion

### Increased genomic instability in HD cells

**Fig 1:**
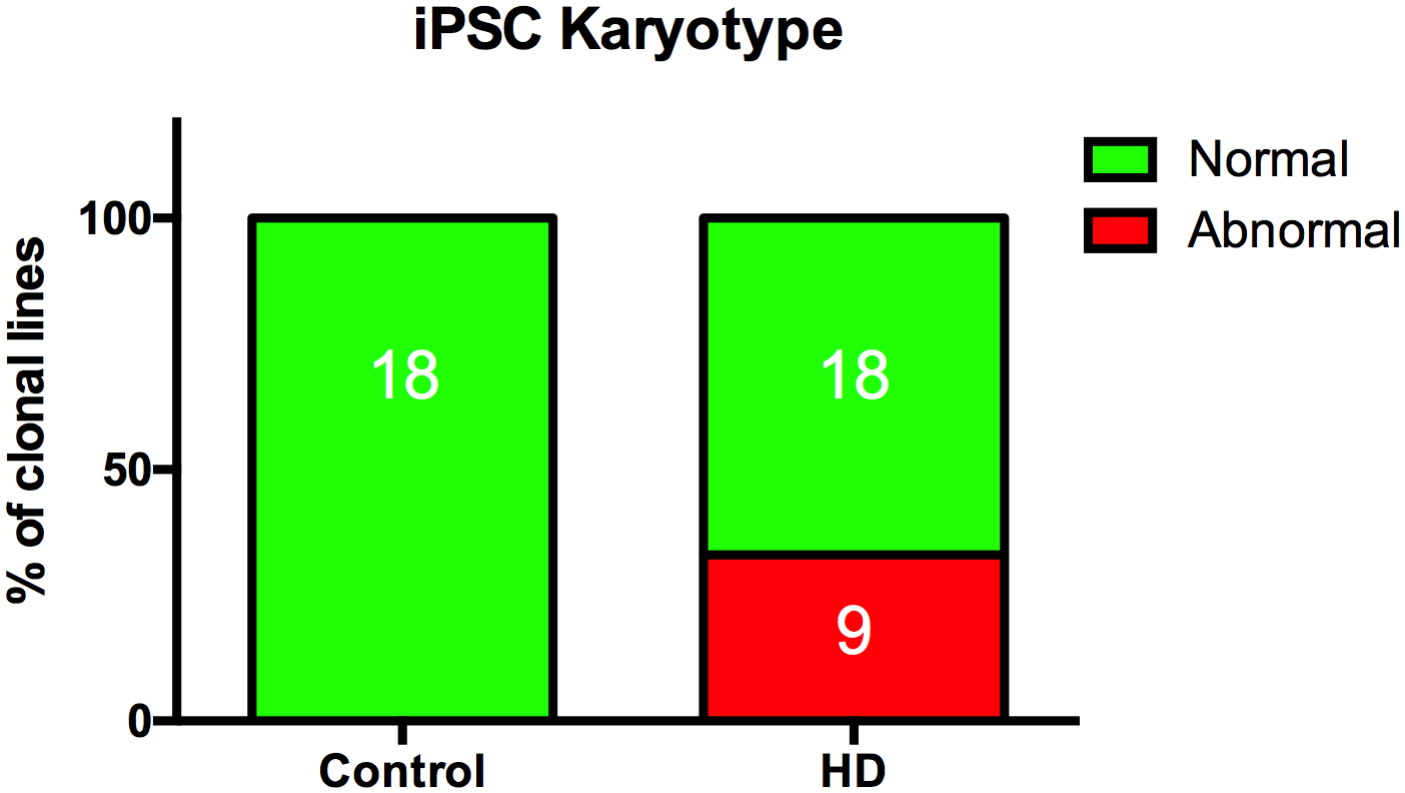
HD iPSC lines have greater propensity for genomic abnormalities than control lines. Control lines from 5 individuals (18 total lines) and HD lines from 5 patients (27 total lines) were analyzed by G banding for chromosomal abnormalities. For detailed karyotype results for each iPSC line in this data set, see Table 1.

We generated iPSC lines from 5 control subjects and 5 patients with HD with a wide range of CAG repeat lengths (35–180 repeats). Karyotype analysis revealed a significantly higher number of HD iPSC lines with genomic abnormalities (9/27 lines from 5 patients) when compared to control lines (Fig 1 and Table 1) and was observed for at least one line in 4 of the 5 patients. All our control lines were normal (0/18 abnormal lines from 5 control subjects) (Fig 1 and Table 1). Using the Chi-square test, disease state was a significant predictor of genomic instability (p = 0.0067). Removing the HD35 and HD57 data from this analysis since these lines were reprogrammed under slightly different conditions (see Methods section) did not change the significance of the outcome (p = 0.0029 by Ch-square test). Furthermore, the length of the longest CAG repeat was significantly correlated with genomic instability in the iPSC lines (all controls were set to 20 CAG repeats [median length from health controls is between 17–19 repeats][17]) with p = 0.006 by binary logistic regression (dropping HD35 and HD57 had no effect, p = 0.032). For comparison, neither age, sex, or passage number correlated with increased risk of genomic instability (p = 0.231 for age, p = 0.853 for sex, p = 0.290 for passage number) (data for HD70 and C-ESS were excluded from age analysis and rerun with all other variables in the model because the age at biopsy was not available.

**Table 1:** Karyotype data for control and HD iPSC lines.

iPSC Line	Patient Phenotype	Abnormal/Total	Age at biopsy	Sex	Passage #	Nature of Abnormalities
CA-11	Control	Normal	25	M	>4	
CA-24	Control	Normal	25	M	9	
CA-26	Control	Normal	25	M	6	
CA-30	Control	Normal	25	M	8	
CC-1	Control	Normal	17	F	7	
CC-2	Control	Normal	17	F	6	
CC-3	Control	Normal	17	F	28	
CC-5	Control	Normal	17	F	7	
CD-2	Control	Normal	53	M	6	
CD-3	Control	Normal	53	M	6	
CD-10	Control	Normal	53	M	9	
CD-12	Control	Normal	53	M	9	
CE-2	Control	Normal	20	F	8	
CE-3	Control	Normal	20	F	7	
CE-4	Control	Normal	20	F	8	
CE-6	Control	Normal	20	F	6	
C-ESS-6	Control	Normal	NA	F	19	
C-ESS-10	Control	Normal	NA	F	26	
***HD35-2***	***HD***	***20/20***	***72***	***F***	***4***	***Inv(5p13q32)***
HD35-5	HD	Normal	72	F	4	
HD35-7	HD	Normal	72	F	4	
HD35-9	HD	Normal	72	F	4	
HD57-1	HD	Normal	19	M	4	
HD57-4	HD	Normal	19	M	4	
HD57-6	HD	Normal	19	M	4	
HD57-7	HD	Normal	19	M	4	
HD57-15	HD	Normal	19	M	4	
***HD58-1***	***HD***	***2/20***	***20***	***M***	***7***	***47*,*XY+12***
HD58-3	HD	Normal	20	M	6	
***HD58-13***	***HD***	***4/20***	***20***	***M***	***17***	***Iso(20q10) [2]; Idic(20q13.3) [2]***
HD58-19	HD	Normal	20	M	10	
HD58-20	HD	Normal	20	M	7	
***HD58-21***	***HD***	***5/20***	***20***	***M***	***10***	***47*,*XY+12***
HD58-31	HD	Normal	20	M	6	
HD58-34	HD	Normal	20	M	6	
HD70-2	HD	Normal	NA	F	~14	
***HD70-5***	***HD***	***1/20***	**NA**	***F***	***~15***	***47*,*XX+12***
HD70-11	HD	Normal	NA	F	8	
HD180-1	HD	20/20	6	M	~15	Del(13q22q32)^a^
***HD180-3***	***HD***	***9/20***	***6***	***M***	***~15***	***47*,*XY+18***
HD180-4	HD	Normal	6	M	6	
HD180-6	HD	Normal	6	M	11	
***HD180-10***	***HD***	***20/20***	***6***	***M***	***6***	Del(13q22q32)*; ***Add(21q22); R(21p11*.*2q22)***
***HD180-14***	***HD***	***20/20***	***6***	***M***	***6***	***Add(Yq11*.*22)***
***HD180-16***	***HD***	***20/20***	***6***	***M***	***6***	***T(1;15)(p10;p10)***

20 metaphase spreads were analyzed for each line. Lines are deemed abnormal if they contain one inversion, deletion, or translocation in one or more cells. Lines are also considered abnormal if they have two or more cells with the same trisomy or monosomy, or 1 cell with a trisomy that commonly occurs in iPSC culture (e.g. trisomy 12). Abnormal lines are in bold and italics. Abnormalities are listed by accepted nomenclature with brackets around the number of cells with a particular abnormality if more than one occurs in a given line.

^a^ If an abnormality was also seen in the parent fibroblast analysis, the iPSC line was considered normal. NA indicates that the information is not available.

**Fig 2:**
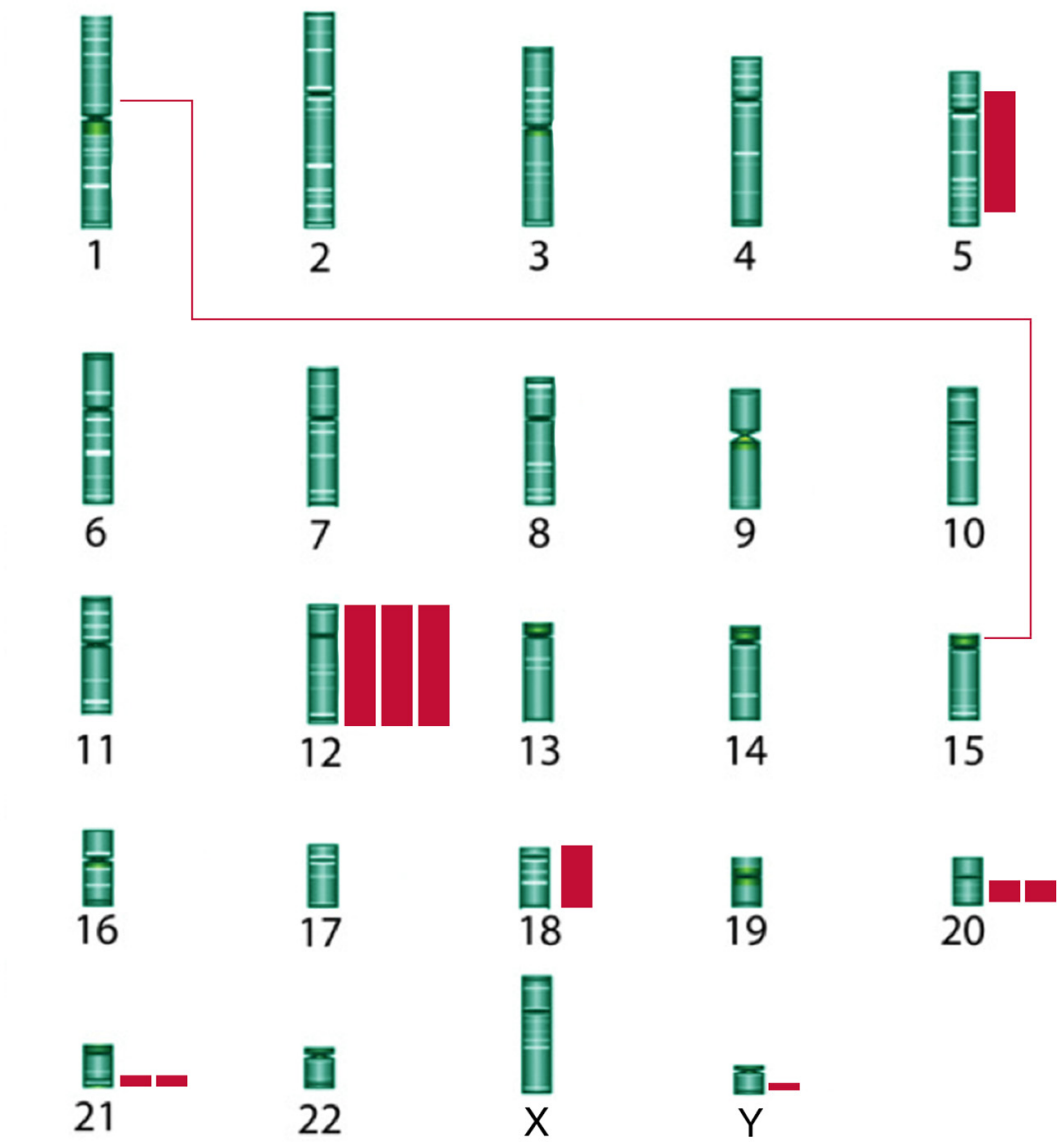
HD iPSC genomic abnormalities. The abnormalities are depicted on a karyogram. Additional chromosomal material and inversions are depicted as solid bars, and translocations as lines connecting the translocated regions. The frequency by which a specific abnormality occurred is indicated (i.e., 3 bars next to chromosome 12 represent 3 lines with trisomy 12).

The HD iPSC genomic abnormalities included trisomies (4), translocations (1), deletions (2), additions [insertions with unknown donor chromosome] (2), inversion (1), isochromosome (1), isodicentrism (1), and ring formation (1) (Table 1). The most frequent abnormality was trisomy 12 (3), which is known to provide a growth advantage for these cells [14]. The HD chromosomal abnormalities identified are depicted on a karyogram in Fig 2 and listed in Table 1. A representative abnormal metaphase spread for each abnormal line is provided in S1 File.

### Parental fibroblast lines of control and HD patients show no differences in the frequency of karyotype abnormalities

We performed karyotype analysis in a subset of parental fibroblast lines to assess if the observed karyotype abnormalities in the HD iPSC lines arose in the fibroblast culture or *de novo* during or after the reprogramming process. We performed a more stringent analysis using 100 cell spreads (instead of 20) for each fibroblast line to minimize the possibility that the abnormalities observed in the iPSC lines resulted from rare genomic events in the fibroblasts that provided an advantage in the reprogramming process. Except for HD180, all assessed fibroblast lines (CA, CC, HD58 and HD70) had similarly low levels of genomic abnormalities (1–2 cells out of 100) (Table 2). The HD180 fibroblast line contained 49 abnormal cells out of 100 with 3 different abnormalities of all different classes (deletion [3 cells], translocation [4 cells], and addition [42 cells]). Although all of the fibroblast lines contained a small number of cells with genomic abnormalities (1-2/100), only one abnormality in the HD180 fibroblasts (del(13)(q22q32) from HD180) was also identified in the iPSC lines (HD180-1 and HD180-10). Thus, HD180-1 was considered normal because the abnormality arose in the fibroblast culture, and HD180-10 was considered abnormal because it contained 2 other abnormalities that did not occur in the fibroblast culture (add(21)(q22),r(21)(p11.2q22) (Table 1). These results indicate that the vast majority of iPSC genomic abnormalities arose either during the reprogramming process or in the iPSC state.

**Table 2:** HD180 fibroblasts have a greater rate of abnormal karyotypes than control and HD58 and HD70 fibroblasts.

Fibroblast Line	Abnormal/Total	Passage #	Nature of Abnormalities
CA	1/100	7	Del(2q33)
CC	1/100	< 20	Add(4q)
HD58	2/100	7	45,X [2]
HD70	2/100	14	Del(8q10) [1]; Del(18p10) [1]
HD180	49/100	17	Del(13q22q32) [3]; T(3;14)(p13;q11.2) [4]; Add(4q23) [42]

100 metaphase spreads from each fibroblast line were analyzed for chromosomal abnormalities.

### Once established, euploid HD and control iPSC lines have similar rates of genomic instability

To assess if the genomic instability observed in HD iPSC lines arises during reprogramming or is due to a genomic instability of the HD iPSC lines, we “aged” 3 control and 4 HD lines by subjecting them to 26–33 passages (an average of 16 additional passages from the original analysis). Control (1/3) and HD (2/4)iPSC lines demonstrated a comparable low frequency of abnormalities (average of 1.6 out of 100) not observed in the line during the original analysis (Table 3). We then analyzed the effect of passage number on genomic instability after adding the data from these aged cell lines to our regression analysis as a covariate with repeat length. Using the entire data set, passage number did not significantly affect the outcome of karyotype analysis (p = 0.077). Our results implicate the reprogramming process as the most likely time point for the development of the karyotypic abnormalities in the HD iPSC lines. The rapid cell divisions and massive restructuring to the cells chromatin and epigenetic markers may contribute to the increased propensity for such abnormalities to occur. In addition, the genetic knockdown of p53 during reprogramming may also have enhanced the frequency of genomic instability in HD cells by reducing DNA damage response elements unmasking an elevation in DNA damage.

**Table 3:** Euploid HD iPSC lines (e.g. lines with a normal karyotype when first generated) accumulate abnormalities at the same rate as euploid control lines.

iPSC Line	Patient Phenotype	Abnormal/Total	Passage #	Nature of Abnormalities
*CA-30*	*Control*	*9/100*	*26(+18)*	*Iso(20)(q10) [8]*
CC-3	Control	0/100	26 (+20)	
CC-3	Control	0/100	32 (+26)	
CD12	Control	0/20	18 (+9)	
HD58-19	HD	0/100	26 (+16)	
HD70-2	HD	0/100	26 (+12)	
*HD70-2*	*HD*	*2/100*	*32 (+18)*	*47,XX,+12 [1]; 47,XX,+22 [1]*
*HD70-5*	*HD*	*3/100*	*33 (+7)*	*47,XX,+12 [2]; Iso(20)(q10) [1]*

The iPSC lines were aged in culture for an additional 7–24 passages after the original karyotyping was performed (see Table 1). 100 metaphase spreads from each iPSC line were analyzed for chromosomal abnormalities.

### HD cells show improved viability and enhanced DNA damage signaling following neocarzinostatin exposure

Although both ESC and iPSC models of Huntington’s disease have been reported by several groups, elevated genomic instability in HD cells has not been previously reported; however, one study reported that 3 out of 6 HD iPSC lines generated where genomically abnormal [18]. Here we report the generation of HD iPS lines using a commonly used episomal based reprogramming method that includes a p53 shRNA knockdown [15], while HD iPSC lines generated by other groups have not utilized an approach that includes p53 knockdown [18–22]. Since p53 is known to suppress iPSC generation, p53 knockdown via shRNA has been used to increase reprogramming efficiency [23], which comes at the cost of decreasing the genomic integrity of iPSC clonal lines generated [24].

**Fig 3:**
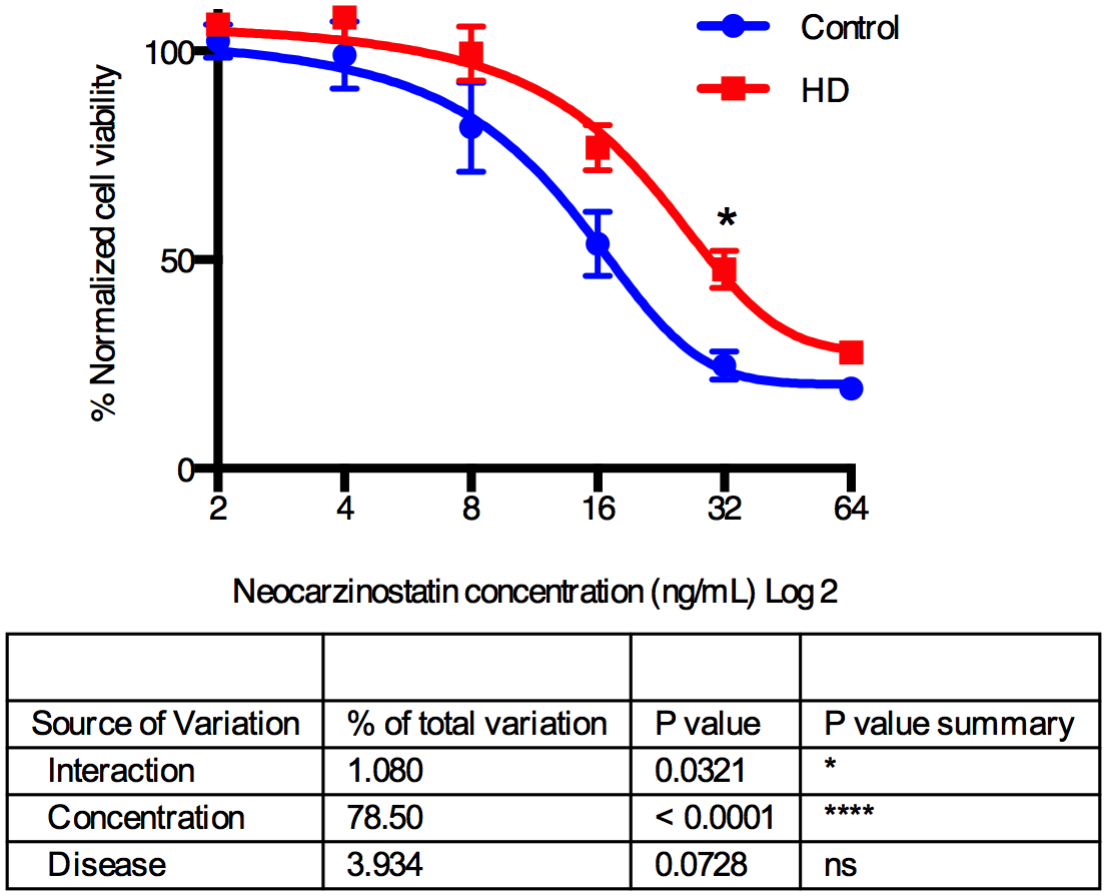
Control and HD iPSCs show significant disease*treatment effect in viability or mitochondrial reductase activity by MTT assay following neocarzinostatin exposure. Karyotypically normal control and HD iPSCs were treated for 1 hour with neocarzinostatin followed by 23 hours of recovery time. The MTT assay was then used to quantify cell viability and/or mitochondrial reductase activity. N = 7 for control (CC-1 = 3, CD-2 = 3, CE-6 = 1) and N = 8 for HD (HD58-19 = 2, HD70-2 = 3, HD180-4 = 3). Points = mean ± SEM. Statistical analysis was performed using repeated measures ANOVA followed by Sidak post-hoc test. * represents p < 0.05.

At first glance, our observation of increased genomic instability in HD iPSC lines seems counter-intuitive to the fact that HD patients have decreased risk for all forms of cancer risk [12]. However, we hypothesize that a slight elevation of DNA damage could result in greater repair and survival compared to controls, but in the context of p53 knockdown this hormesis response is negated resulting in elevated genomic instability. To test the first part of this hypothesis, we treated HD and control iPSCs with neocarzinostatin, a compound known to generated double stranded DNA breaks in cells. Using the MTT cell viability assay, we determined neocarzinostatin treated HD iPSCs have significantly better survival with increasing levels of neocarzinostatin (HD*neocarzinostatin interaction effect [p = 0.0321] and p < 0.05 for post-hoc t-test at 32 ng/mL neocarzinostatin using repeated measures ANOVA) (Fig 3). Therefore, this hormesis response could lead to the decreased cancer risk observed in patients.

**Fig 4:**
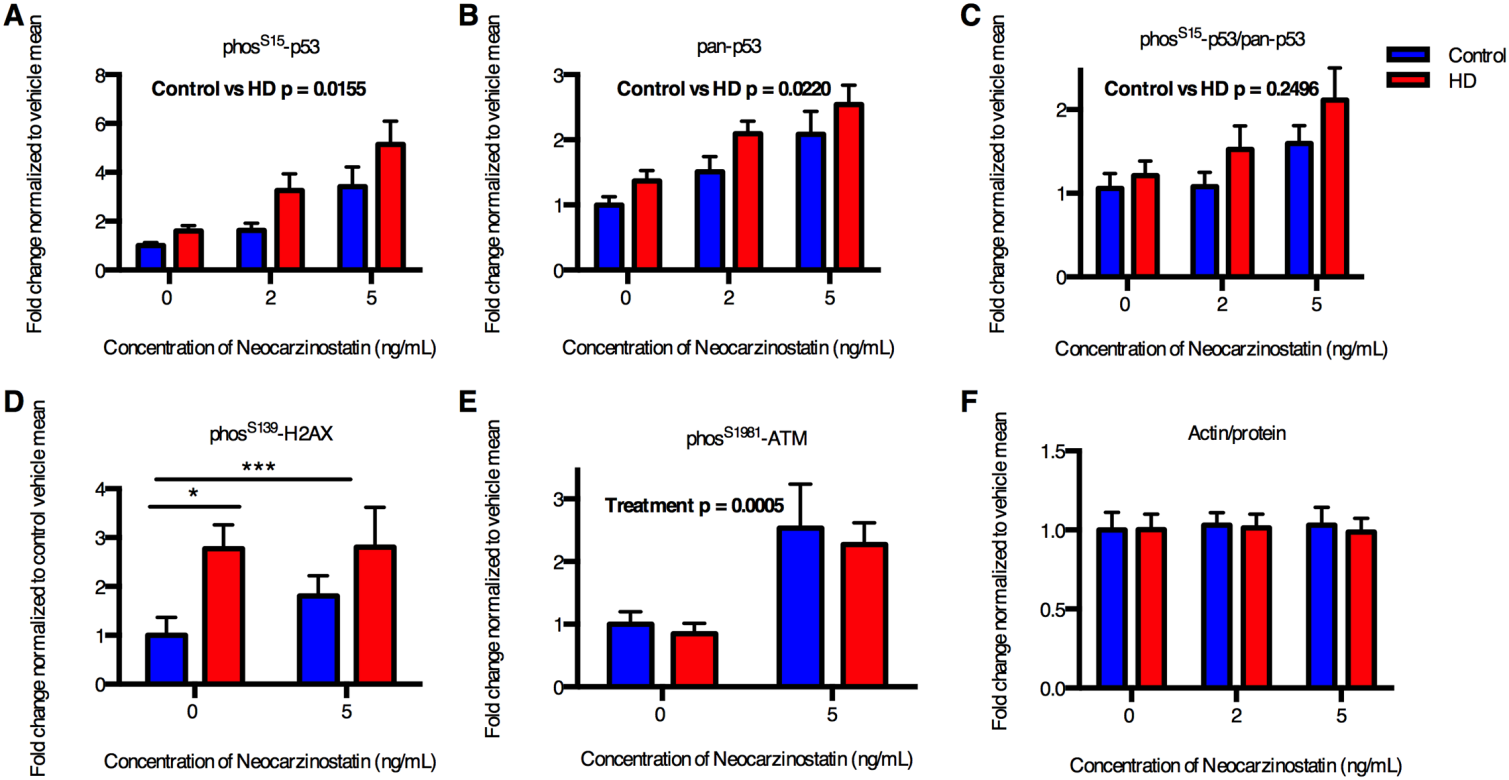
Karyotypically normal HD iPSCs have elevated basal and DNA damage dependent ATM-p53 signaling. Control and HD iPSCs were treated for 1 hour with DNA damaging agent, neocarzinostatin. Western blot analysis was performed for phosphorylated p53 at serine 15 (**A**), total p53 (**B**), phosphorylated histone H2AX at serine 139 with neocarzinostatin treatment (**D**), and phosphorylated ATM at serine 1981 under basal conditions (**E**). The ratio of phosphorylated to total p53 is plotted in (**C**) and a ratio of actin to the total protein normalization in (**F**). N = 5 for control (CC-1 = 3, CE-6 = 2) and 6 for HD (HD70-2 = 3, HD180-4 = 3). Bars = SEM. P value is calculated via 2-way Repeated Measure ANOVA (**A, B, C, E**) or t-test (**D**).

The mechanism behind this hormesis could be elevation of p53 signaling. Elevated p53 has previously been shown to be protective in the context of ischemic preconditioning [25], and elevated p53 signaling is one of the most consistent phenotypes across HD disease models, often observed with concurrent elevation of either H2AX or ATM phosphorylation, indicating an ATM kinase-dependent pathway activation of p53 [1, 4, 8, 9, 26, 27]. Elevations in ATM-p53 pathway could indicate an increase in DNA damage in HD cells under basal conditions. Therefore, we quantified ATM-p53 signaling in karyotypically normal HD and control iPSCs under basal and DNA damaging conditions (neocarzinostatin treatment at concentrations [2–5 ng/mL] that do not induced cell death [Fig 3]), which activates the ATM-p53 pathway. We observed a significant increase in the phosphorylation of p53 at serine-15 and total p53 expression in HD iPSCs compared to controls across the data set (Fig 4A–4C). Neocarzinostatin increased p53 phosphorylation and total p53 protein in both control and HD cell lines at concentrations that do not induce lose of cell viability/mitochondrial toxicity (Fig 3). An example western blot for neocarzinostatin-induced p53 phosphorylation is shown in S2 Fig. We confirmed activation of DNA damage responses at these low concentrations of neocarzinostatin by measuring the phosphorylation of ATM at serine 1981, a direct response to double stranded DNA breaks [28] (Fig 4E). The relative differences in p53 and phosphorylated p53(S15) between HD and control cells was similar in neocarzinostatin and untreated cells (Fig 4). Normalization of phosphorylated p53(S15) and p53 protein by the untreated levels for each genotype revealed similar fold-changes in neocarzinostatin induced phosphorylated p53(S15) and total p53 levels between control and HD. Consistent with a basal elevation in p53 phosphorylation, the HD iPSCs also showed basal elevation of H2AX(S139) phosphorylation (Fig 4D), a marker of double-stranded DNA damage that is also downstream of the ATM kinase DNA damage response pathway. These data indicate either an increase in the degree of DNA damage itself and/or the sensitivity of DNA damage response pathways, even in the absence of neocarzinostatin. We also compared our normalization factor (total protein by gel staining) to actin band intensity to validate our methodology (Fig 4F).

**Fig 5:**
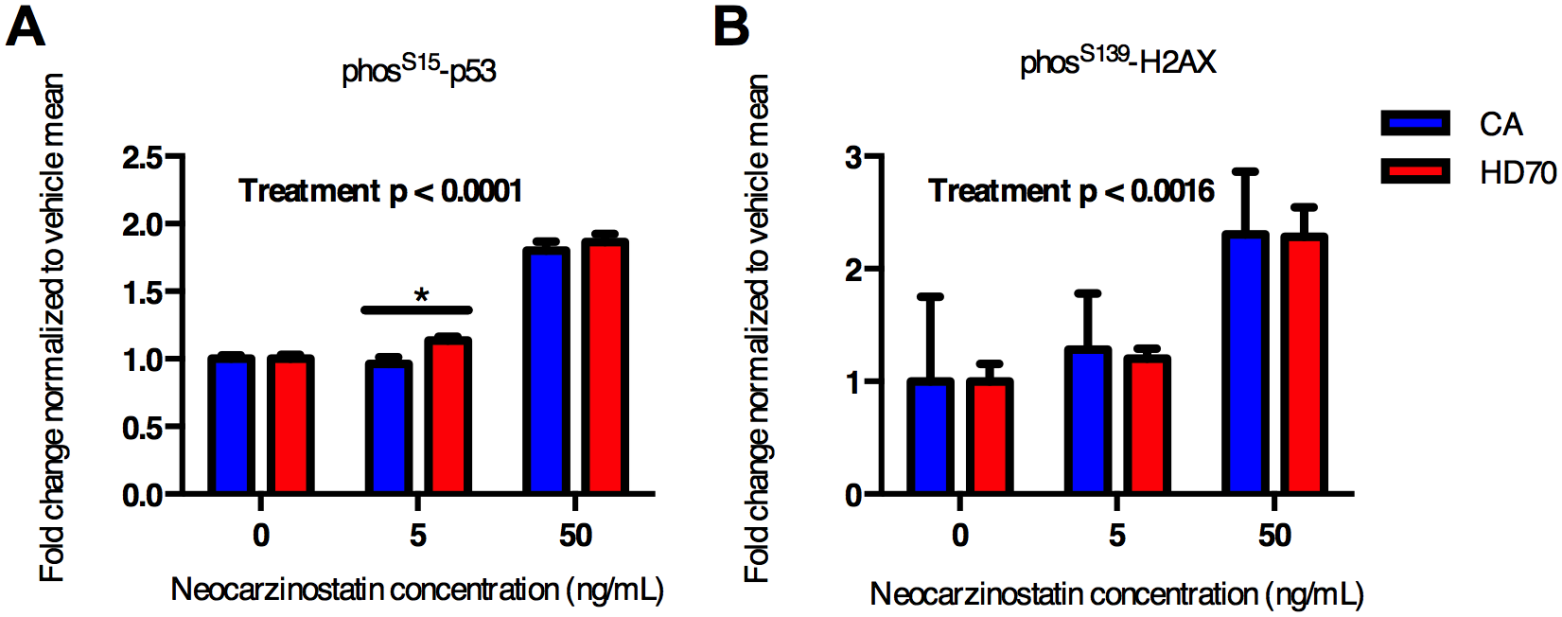
HD fibroblasts have elevated DNA damage dependent p53 phosphorylation. Control (CA) and HD (HD70) fibroblasts were treated for 1 hour with DNA damaging agent neocarzinostatin. In-Cell Western analysis was performed for phosphorylation of p53 at serine 15 (**A**) and H2AX at serine 139 (**B**). N = 3 independent experiments. Analysis was performed using repeated measure ANOVA with Sidak host-hoc test. P < 0.05 considered significant, * represents p < 0.05.

The HD fibroblasts were also tested for elevated p53 and H2AX phosphorylation in response to neocarzinostatin treatment. Using the In-Cell western assay, neocarzinostatin treatment increased the phosphorylation of both proteins (Fig 5). At 5 ng/mL neocarzinostatin, HD70 had significantly elevated p53 phosphorylation while the control (CA) remained at basal levels (Fig 5A). This is consistent with reports of elevated ATM signaling activity in HD cellular models in response to oxidative stress [8]. H2AX phosphorylation levels did not show a disease specific difference; however, this could be due to the much greater variability in this data compared to phospho-p53.

### No evidence for HD-dependent centrosome amplification or chromosomal segregation dysfunction

DNA damage and repair problems in HD cells have been previously identified. Expression of mutant Huntingtin can cause increased DNA DSBs in cells and mouse brain [5, 9, 10]. Evidence from mouse R6/2 HD model suggested increased DNA oxidation as a key HD disease mechanism [29, 30]. However, studies measuring oxidized DNA in HD patient postmortem brains have provided conflicting results [31–33]. Chromosomal abnormalities can occur by DNA damage; DNA double-stranded breaks (DSBs) can be erroneously repaired to cause translocations, other rearrangement events (i.e. deletions, inversions), and possibly aneuploidy. HD cell and animals models have shown increased DNA-damage and DNA-damage induced signaling responses (ie. ATM-p53) [1, 4, 5, 9, 10, 27, 29, 30, 34]. Alternatively, these genomic aberrations, particularly aneuploidy, can arise from dysregulated chromosomal segregation during cell division [35]. HD cells have shown disruption to centrosomes and mitotic spindles that could potentially lead to misappropriation of chromosomes during mitosis [36–38]. One of these studies identified increased centrosomal amplification in mouse and HD fibroblasts [38]. However, we found no significant difference in the number of centrosomes in HD and control iPSCs as measured by counting pericentrin foci (S3 Fig). Furthermore, we found no evidence of a disease-dependent cell cycle arrest or disruption, which suggests that HD does not cause dysregulated chromosomal segregation (S4 Fig). An example cell cycle histogram is provided in S1 Fig. Our cell cycle arrest study does indicate that neocarzinostatin exposure caused an increased arrest in G_0_/G_1_ and S phases but no significant differences by genotype. Therefore, we do not believe that either centrosomal amplification or mis-segregation are significant processes in the selective HD-dependent genomic instability in our model. Future experiments to address the mechanism of instability in the HD reprogramming should likely focus on ATM-p53 dependent pathways given (1) the known alterations in ATM-p53 signaling that have been reported in HD [1, 4, 5, 9, 10, 27, 29, 30, 34], (2) that p53 is knocked down during the reprogramming process that we utilized, while other groups in which reprogramming did not include p53 knockdown did not report increased genomic instability [18–22], and (3) loss of p53 pathway has been reported to increase rates of genomic instability during reprogramming generally [24].

## Conclusions

Our data demonstrate increased genomic abnormalities in iPSC lines reprogrammed from fibroblasts of patients with HD versus control subjects. This was in the context of a common reprogramming approach in which p53 is knocked down by an shRNA. Additionally, the karyotypically normal HD iPSC lines have elevated DNA damage signaling compared to controls specifically in total p53 levels and levels of p53 and H2AX phosphorylation. HD fibroblasts and neural progenitors differentiated from the iPSCs also demonstrate an elevation in phospho-p53 response to DNA damage (Fig 5 and reference [11]). We also provide evidence that suggests the genomic instability occurs during the reprogramming process, the time in which the cells were expressing the p53 shRNA. Taken together these data support existing evidence for increased DNA damage and DNA damage response in HD. However, one caveat of this study is that it does not include a comparison of reprogramming for isogenic fibroblasts (with and without the HD CAG expansion). Thus, it is formally possible (but unlikely) that other genetic differences between our 5 control fibroblasts and our 5 HD fibroblasts are also contributing to these differences.

The increased genomic instability [12] during reprogramming is likely due to p53 knockdown. P53 activity itself inhibits reprogramming to the pluripotent state, hence p53 knockdown increases efficiency of reprogramming [15, 24, 39]. Normally, transient p53 knockdown, as is achieved by the reprogramming method we employed, does not irreparably increase genomic instability [15, 39] and reprogramming can be achieved without an excessive rate of karyotypic abnormalities as seen in our non-HD reprogramming (Fig 1 and Table 1). However, we hypothesize that the decrease in p53 activity that is needed to achieve reprogramming (either stochastically, or as a matter of design during the reprogramming process) selectively increases the risk of genomic instability in the presence of an HD genotype by inhibiting a potential p53-mediated hormesis effect resulting from low-level HD dependent DNA damage. This suggests that the elevated p53 activity that we, and others, have seen in HD cells may in fact be necessary to maintain a normal karyotype in these cells. Unfortunately, the low efficiency (0.01%) and long time needed for reprogramming (3 weeks) does not easily allow for further investigation into the effects of reprogramming on genomic stability. However, reprogramming in general leads to a high level of replication stress that results in genomic instability [40]. Currently, we do not have an explanation for the elevated DNA damage response in HD cells under basal conditions; however, one group found increased DNA damage due to the expression of mutant Huntingtin protein [10]. The elevation in p53 signaling may play an important role in HD pathology and warrants further investigation. Our data implicate an influence of CAG repeat expanded mutant *Huntingtin* on DNA damage response pathways during the process of reprogramming human fibroblasts to pluripotency as well as in established human pluripotent stem cells.

## Supporting Information

S1 Fig Cell cycle analysis histogram. An example cell cycle histogram is provided for the HD180-4 iPSC line with vehicle or 2 ng/mL neocarzinostatin treatment. 10,000 events were initially measured followed by gating via forward and side scatter.
(PDF)

S2 Fig Example western blot image for iPSCs treated with neocarzinostatin. CE-6 and HD70-2 iPSCs were treated with 0, 2, or 5 ng/mL neocarzinostatin for 1 hour before collection and lysis. Western blots were performed for phospho-p53(S15) and total p53.
(TIFF)

S3 Fig Mutant *Huntingtin* does not alter the incidence of multipolar cells. Karyotypically normal control and HD human iPSCs were stained pericentrin, a protein marker of centrosomes. 20 mitotic cells for each experiment were identified by Hoechst DNA stain with clear chromosomes. Cells were then scored as either multipolar (3+ centrosomes identified by pericentrin) or normal (1–2 centrosomes). N = 6 for each bar (3 each for CA-30, CC-3, HD58-19, and HD70-2). Bars = SEM. P value by t-test.
(TIFF)

S4 Fig Cell cycle analysis shows a neocarizonstatin but not genotype effect. Karyotypically normal control and HD human iPSCs were treated with neocarzinostatin for 1 hour. After 23 hours of recovery, cells were analyzed by flow cytometry with the DNA dye propidium iodide to quantify cellular DNA content, which was used to categorize individual cell phase. N = 2 for control (both CC-1) and N = 4 for HD (2 for HD70-2 and 2 for HD180-4). Statistical analysis performed by paired t-test.
(TIFF)

S1 File Abnormal HD iPSC karyograms. A single representative abnormal metaphase spread for each abnormal line is provided.
(PDF)
