# Supplementary figures and images for: Genomic Instability Associated with p53 Knockdown in the Generation of Huntington’s Disease Human Induced Pluripotent Stem Cells
Source: PLoS One. 2016 Mar 16;11(3):e0150372. doi: 10.1371/journal.pone.0150372 (PMC4794230; doi:10.1371/journal.pone.0150372)

HD180-4 Vehicle

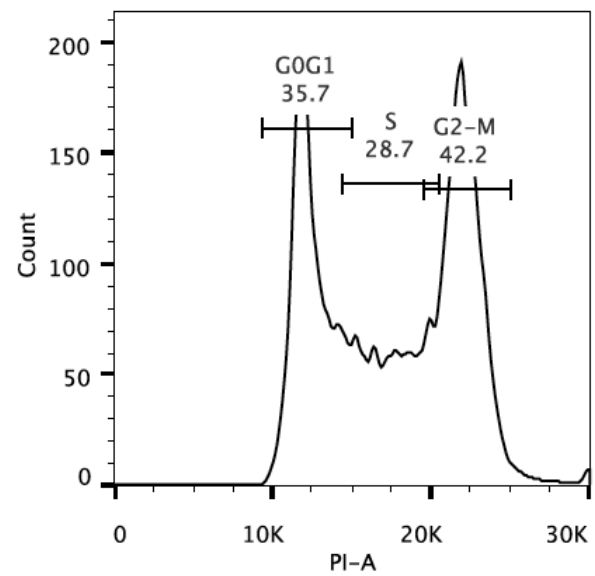

HD180-4 2 ng/mL NCS

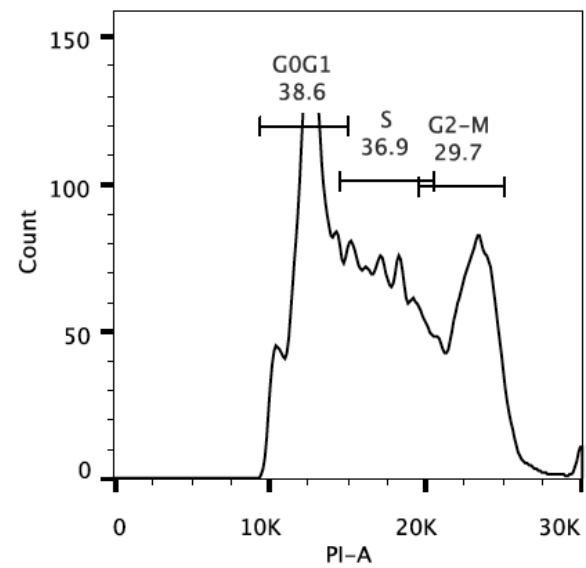

Supplement: S1 Fig — An example cell cycle histogram is provided for the HD180-4 iPSC line with vehicle or 2 ng/mL neocarzinostatin treatment. 10,000 events were initially measured followed by gating via forward and side scatter. (PDF) [file pone.0150372.s001.pdf]

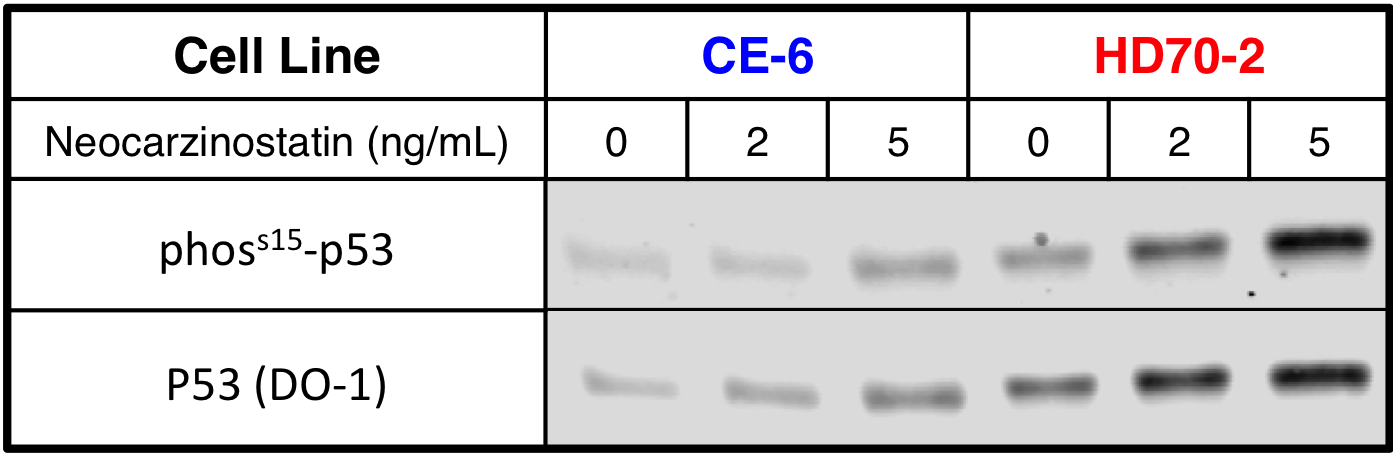

Supplement: S2 Fig — CE-6 and HD70-2 iPSCs were treated with 0, 2, or 5 ng/mL neocarzinostatin for 1 hour before collection and lysis. Western blots were performed for phospho-p53(S15) and total p53. (TIFF) [file pone.0150372.s002.tiff]

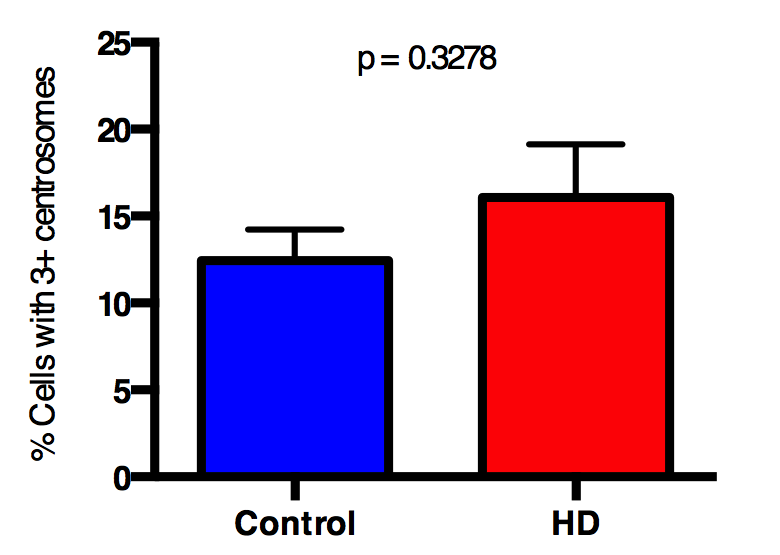

Supplement: S3 Fig — Karyotypically normal control and HD human iPSCs were stained pericentrin, a protein marker of centrosomes. 20 mitotic cells for each experiment were identified by Hoechst DNA stain with clear chromosomes. Cells were then scored as either multipolar (3+ centrosomes identified by pericentrin) or normal (1–2 centrosomes). N = 6 for each bar (3 each for CA-30, CC-3, HD58-19, and HD70-2). Bars = SEM. P value by t-test. (TIFF) [file pone.0150372.s003.tiff]

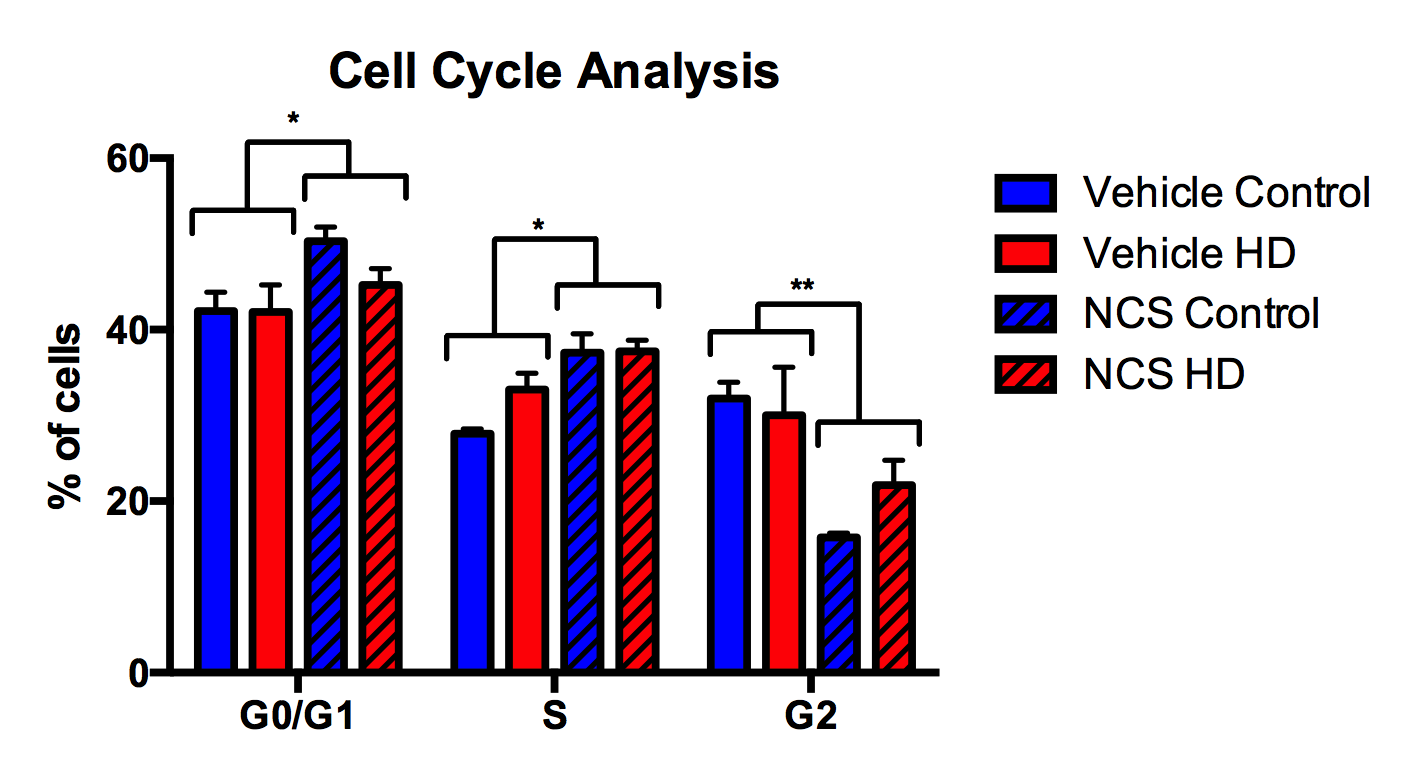

Supplement: S4 Fig — Karyotypically normal control and HD human iPSCs were treated with neocarzinostatin for 1 hour. After 23 hours of recovery, cells were analyzed by flow cytometry with the DNA dye propidium iodide to quantify cellular DNA content, which was used to categorize individual cell phase. N = 2 for control (both CC-1) and N = 4 for HD (2 for HD70-2 and 2 for HD180-4). Statistical analysis performed by paired t-test. (TIFF) [file pone.0150372.s004.tiff]
