# Supplementary material for: Genomic Instability Associated with p53 Knockdown in the Generation of Huntington’s Disease Human Induced Pluripotent Stem Cells
Source: PLoS One. 2016 Mar 16;11(3):e0150372. doi: 10.1371/journal.pone.0150372 (PMC4794230; doi:10.1371/journal.pone.0150372)
Supplement: S1 File — A single representative abnormal metaphase spread for each abnormal line is provided. (PDF) [file pone.0150372.s005.pdf]

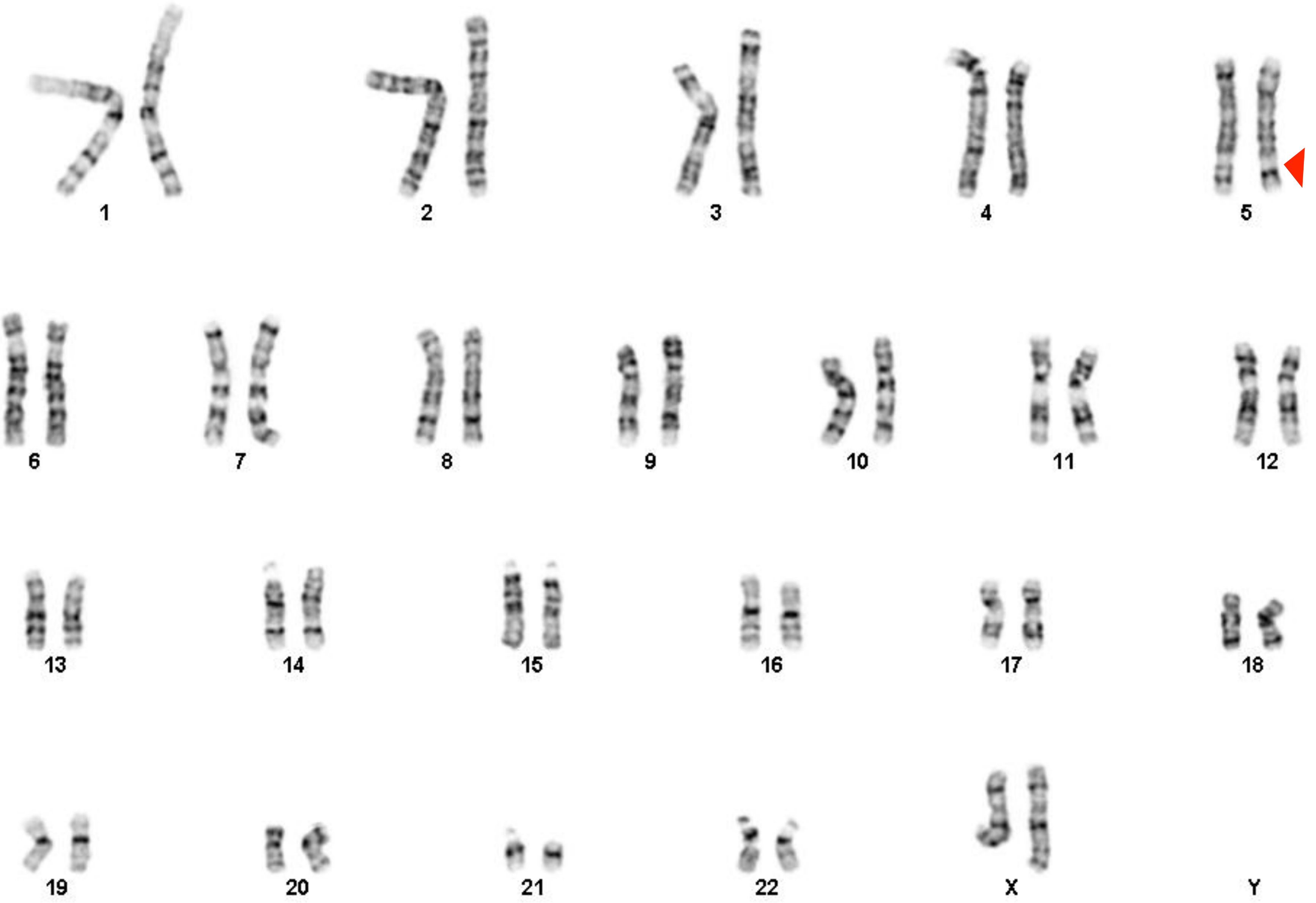

46,XX,inv(5)(p13q32) HD35-2 p4 cell 11 07/08/2014

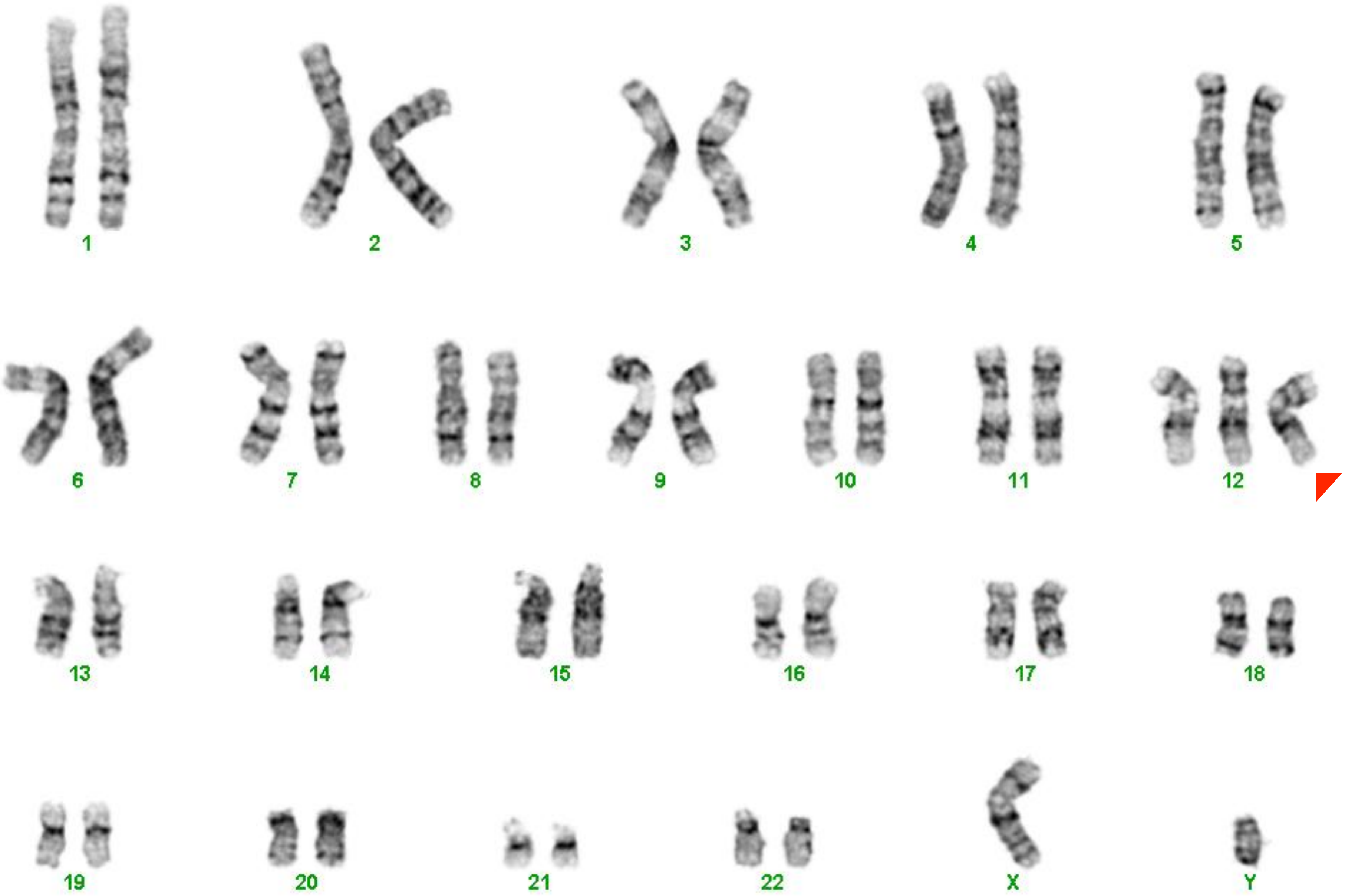

47,XY,+12 HD58-1 Cell 18 11-07-2011

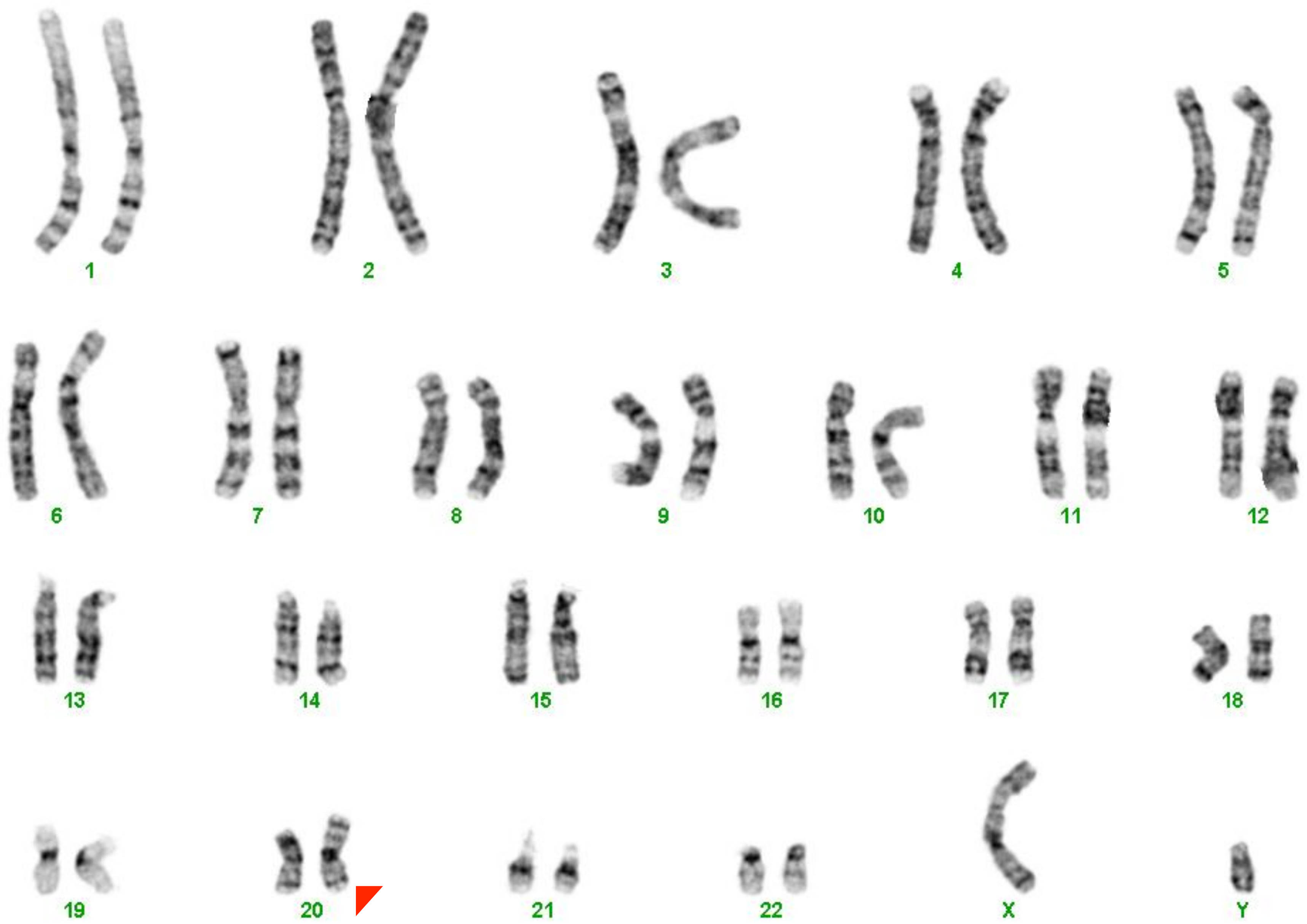

46,XY,i(20)(q10) HD58-13 P17 10-11-2011 Cell 15

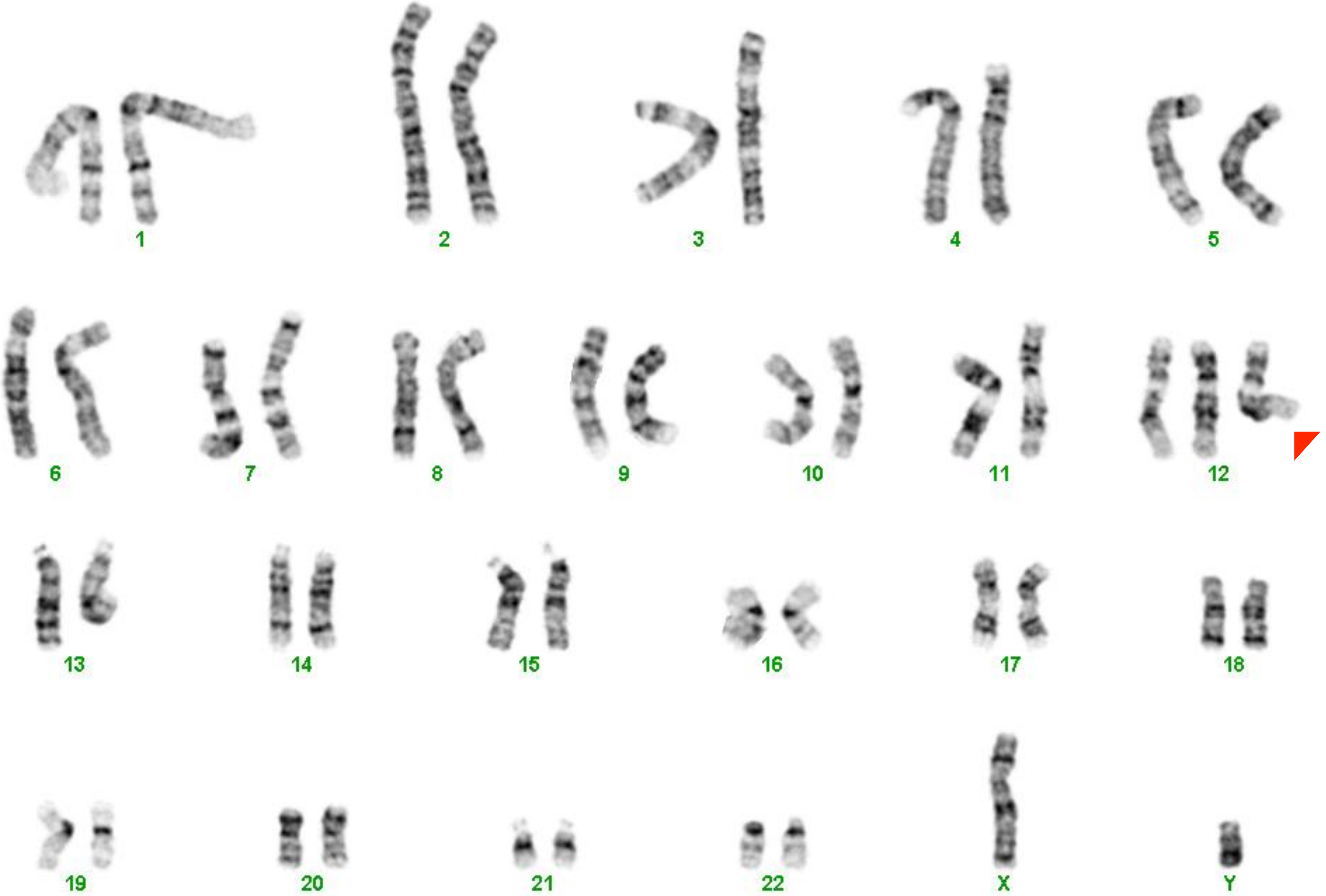

47,XY,+12 HD58-21, P10 Cell 10 rec'd 1-18-2012

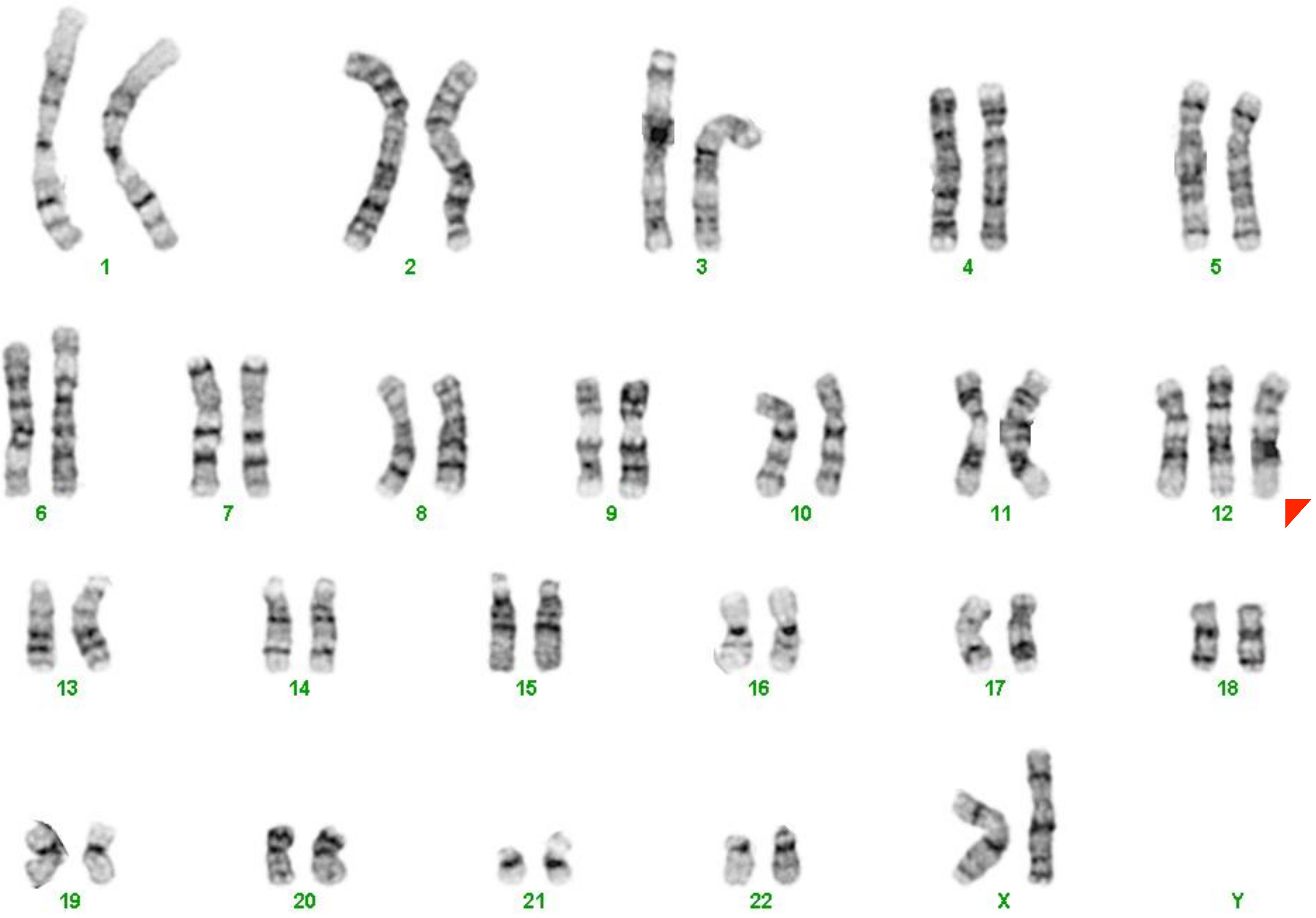

47,XX,+12 HD70-5 P15 09-13-2011 Cell 10

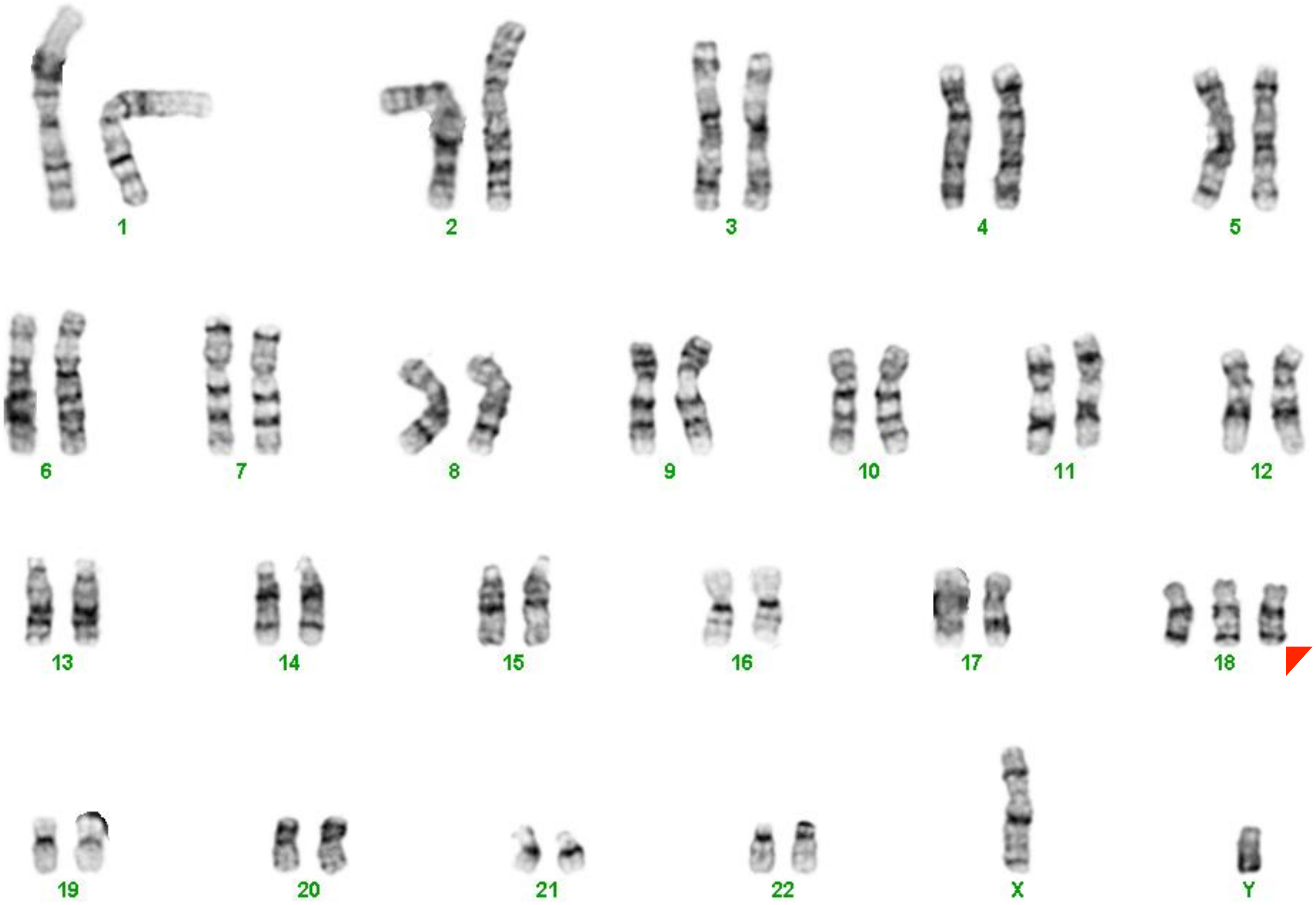

47,XY,+18

09-20-2011 HD180-3 Cell 11

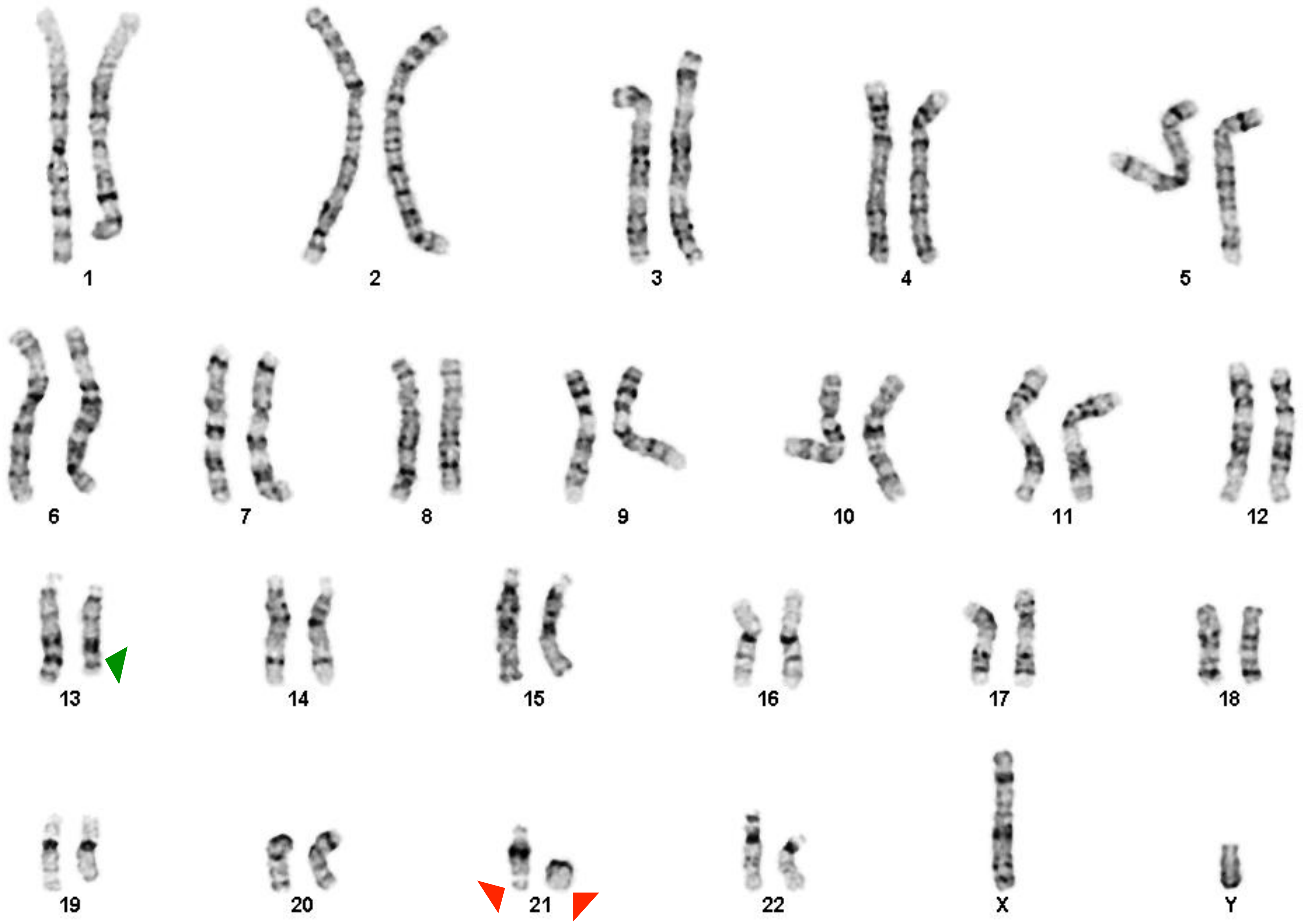

46,XY,del(13)(q22q32),add(21)(q22),r(21)(p11.2q22)   HD180-10 p6 Cell 6   12-12-2012

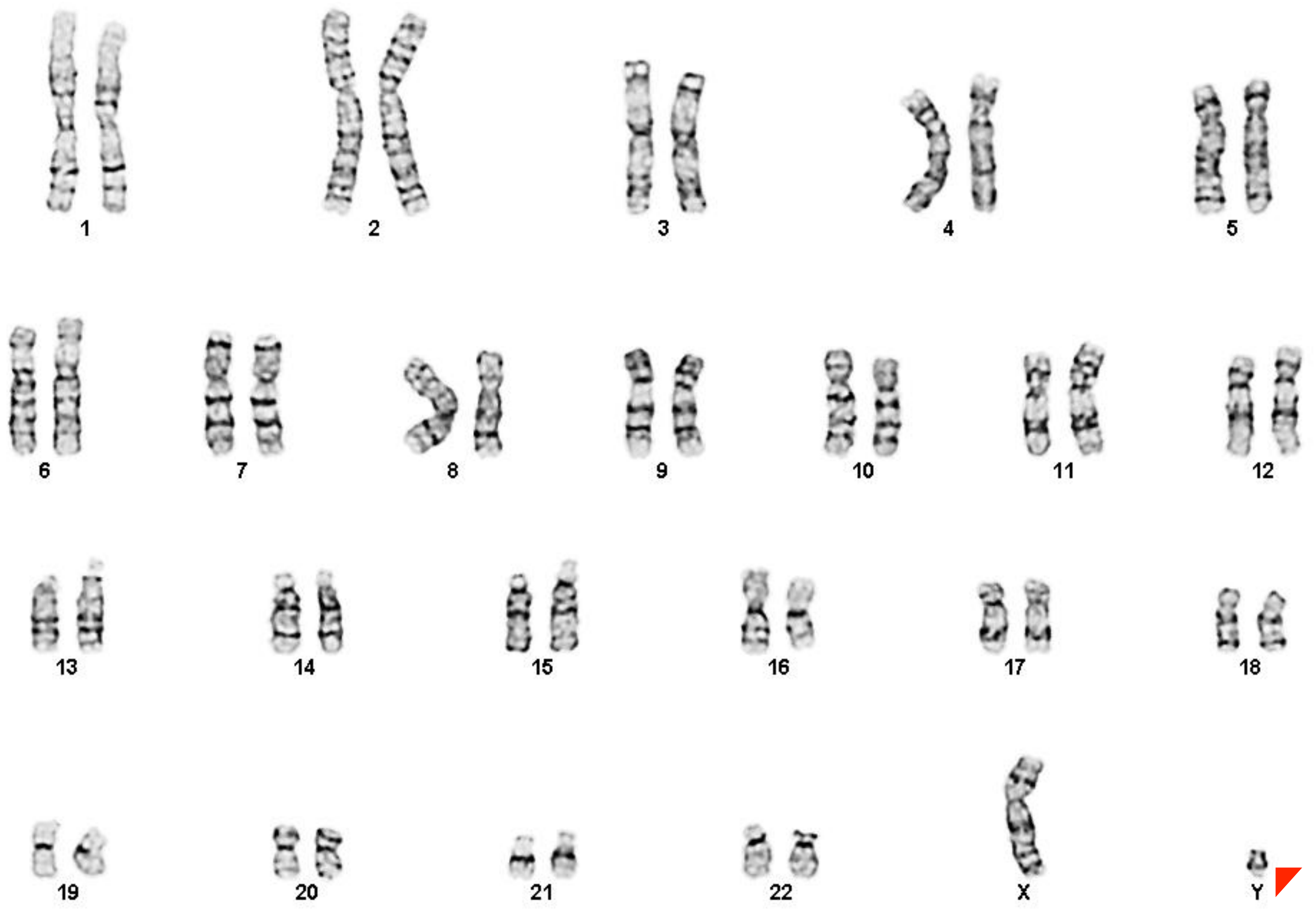

46,X,add(Y)?(q11.22)   HD180-14 p6 Cell 20   12-12-2012

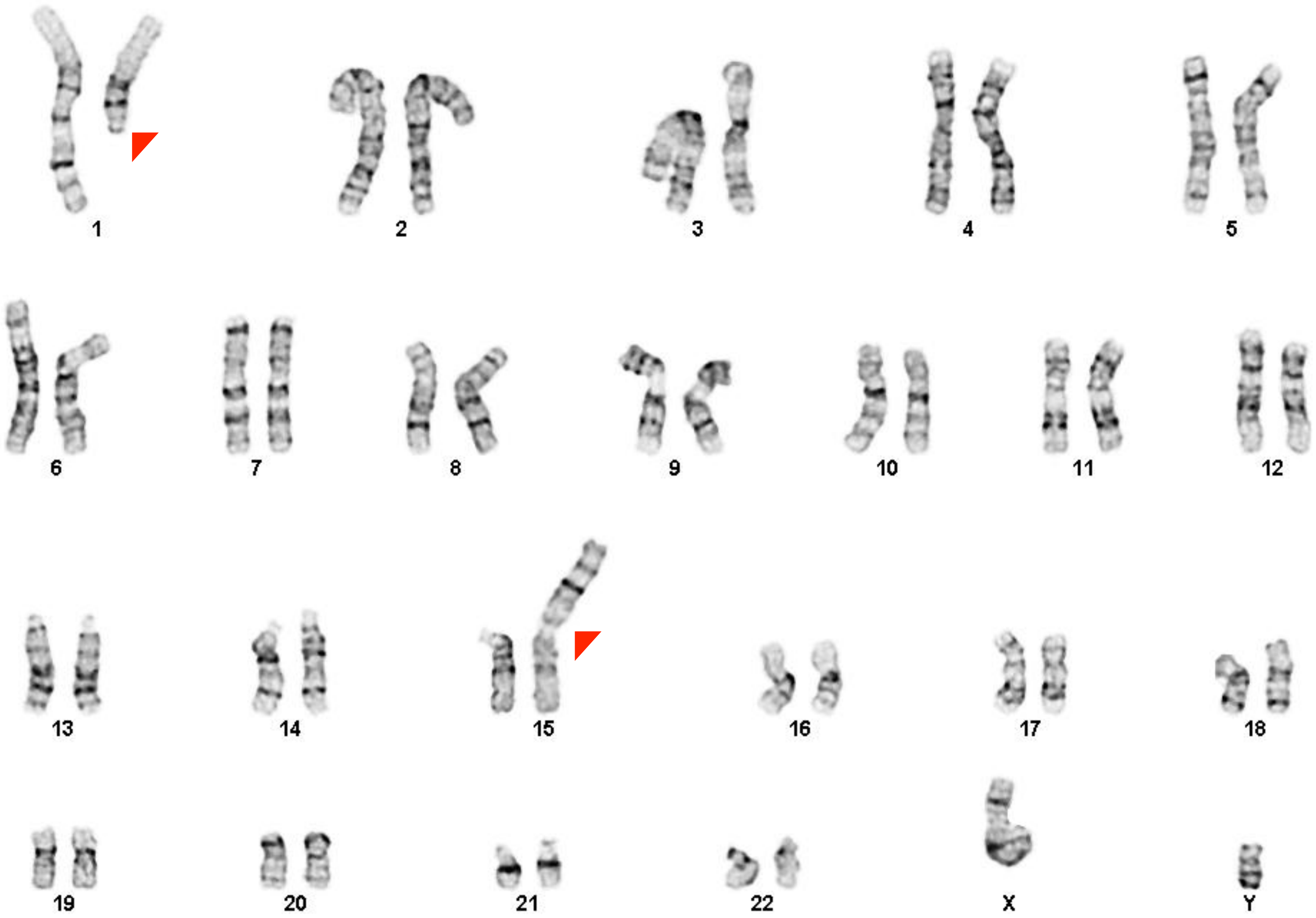

46,XY,t(1;15)(p10;p10) HD180-16 p6 Cell 6 12-12-2012
